# Supplementary material for: All-at-once RNA folding with 3D motif prediction framed by evolutionary information
Source: Nat Methods. 2025 Oct 3;22(10):2094–106. doi: 10.1038/s41592-025-02833-w (PMC12510878; doi:10.1038/s41592-025-02833-w)
Supplement: Supplementary file 3 — Source code to run CaCoFold-R3D. Data and results. [file 41592_2025_2833_MOESM3_ESM.gz › supplemental_material/Figures/Figure_S3.pdf]

#HL	Loop(5'-3')	L(5'-3')	R(5'-3')	name
HL	N	G	RA	GNRA-tetraloop
HL	URA	-	-	U-turn
HL	UNCG	-	-	UNCG-tetraloop
HL	ANYA	-	-	ANYA-tetraloop
HL	CUYG	-	-	CUYG-tetraloop
HL	YGNN	-	-	YGNN-tetraloop
HL	GANC	-	-	GANC-tetraloop
HL	UNAC	-	-	UNAC-tetraloop
HL	URRR	-	-	T-loop-tetraloop
HL	UAACR	-	-	L8_RNaseP_bact_a
HL	AG	UAGUACG	AGGACC	Sarcin-ricin_loop
HL	AGGAY	-	-	CsrA_binding
HL	GAGUA	-	-	GAGUA_pentaloop
HL	AA	-	-	AA-5SrRNA
HL	GCRYA	-	-	U6-loop

#BL	Loop(5'-3')	L(5'-3')	R(5'-3')	name
BL	UGRAA	-	-	Docking-elbow
BL	AA	-	-	AA-bulge
BL	G	-	-	bulged-G

#IL	Loop-L(5'-3')	Loop-R(5'-3')	L-o(5'-3')	R-o(5'-3')	L-i(5'-3')	R-i(5'-3')	name
IL	-	RNN	N,G,A	N,A,R	-	-	K-turn
IL	-	RNN	G,A	A,R	-	-	K-turn-b
IL	G	N	G,A	A,U	U,A	A,G	Loop-E
IL	UAAG	UAU	C,C	G,G	-	-	GAAA_Tetraloop-receptor
IL	CVC	V	-	-	-	-	C-loop
IL	G	-	G,A	A,A	U,A	A,G	G-bulge
IL	G	-	U,A	C,C	U,A	A,G	G-bulge_Das
IL	-	-	G,A	A,G	-	-	Tandem-GA
IL	YCC	AAC	-	-	-	-	Twist-up
IL	YAA	RAN	-	-	-	-	UAA_GAN
IL	RR	YN	R	R	A	Y	J4a/4b
IL	AA	AA	-	-	-	-	J4/5-IL
IL	GUA	GG	-	-	-	-	GUA^GG_RRE
IL	CAGG	AGCA	-	-	-	-	S_domain
IL	AN	-	G,A	A,U	-	-	Hook-turn
IL	UGRAA	-	-	-	-	-	Docking-elbow-IL
IL	UU	AUU	-	-	-	-	pK-turn

#J3	S1(5'-3')	S2(5'-3')	S3(5'-3')	name
J3	N	CUGA	A	J3_hammerhead
J3	U	YUCUAC	AC	J3_purine
J3	NN	NNNNNN	NN	J3_typeA
J3	NNNN	NNNN	NNNN	J3_typeB
J3	NN	NNN	NNNNNN	J3_typeC
J3	N	UGAGA	N	J3_TPP
J3	A	RAA	-	J3_groupII

#J4	S1(5'-3')	S2(5'-3')	S3(5'-3')	S4(5'-3')	name
J4	-	AA	-	U	J4_HCV_IRES
J4	N	-	NNN	-	J4_tRNA
J4	N	N	NN	-	J4_manA
J4	R	-	-	R	J4_U1RNA

#BS	Loop(5'-3')	name
BS	UKNRW	T-loop
BS	RRGU	LoopE-a
BS	RARR	LoopE-b
BS	AAYAARAACAANARR	CRC_binding
BS	AGGAY	CsrA_motif
